# NMDA Receptor Subunits in the Adult Rat Hippocampus Undergo Similar Changes after 5 Minutes in an Open Field and after LTP Induction

**DOI:** 10.1371/journal.pone.0055244

**Published:** 2013-02-01

**Authors:** Maria Veronica Baez, Maria Victoria Oberholzer, Magali Cecilia Cercato, Marina Snitcofsky, Alejandra Ines Aguirre, Diana Alicia Jerusalinsky

**Affiliations:** 1 Instituto de Biología Celular y Neurociencia (IBCN) “Prof. Eduardo De Robertis” CONICET – UBA, Facultad de Medicina, Universidad de Buenos Aires, Buenos Aires, Argentina; 2 CBC, Universidad de Buenos Aires, Buenos Aires, Argentina; Federal University of Rio de Janeiro, Brazil

## Abstract

NMDA receptor subunits change during development and their synaptic expression is modified rapidly after synaptic plasticity induction in hippocampal slices. However, there is scarce information on subunits expression after synaptic plasticity induction or memory acquisition, particularly in adults. GluN1, GluN2A and GluN2B NMDA receptor subunits were assessed by western blot in 1) adult rats that had explored an open field (OF) for 5 minutes, a time sufficient to induce habituation, 2) mature rat hippocampal neuron cultures depolarized by KCl and 3) hippocampal slices from adult rats where long term potentiation (LTP) was induced by theta-burst stimulation (TBS). GluN1 and GluN2A, though not GluN2B, were significantly higher 70 minutes –but not 30 minutes- after a 5 minutes session in an OF. GluN1 and GluN2A total immunofluorescence and puncta in neurites increased in cultures, as evaluated 70 minutes after KCl stimulation. Similar changes were found in hippocampal slices 70 minutes after LTP induction. To start to explore underlying mechanisms, hippocampal slices were treated either with cycloheximide (a translation inhibitor) or actinomycin D (a transcription inhibitor) during electrophysiological assays. It was corroborated that translation was necessary for LTP induction and expression. The rise in GluN1 depends on transcription and translation, while the increase in GluN2A appears to mainly depend on translation, though a contribution of some remaining transcriptional activity during actinomycin D treatment could not be rouled out. LTP effective induction was required for the subunits to increase. Although in the three models same subunits suffered modifications in the same direction, within an apparently similar temporal course, further investigation is required to reveal if they are related processes and to find out whether they are causally related with synaptic plasticity, learning and memory.

## Introduction

NMDA (N-methyl-D-aspartate) receptors (NMDAR) are heterotetramers composed by two GluN1 obligatory subunits and two regulatory subunits: GluN2 (A–D) or GluN3 (A–B) [1]. Most NMDAR contain GluN2 subunits [2], with GluN2A and GluN2B being the major regulatory subunits in the forebrain, particularly in the hippocampus. These two subunits have different pharmacological and biophysical properties [3] and are believed to play a determining role in synaptic plasticity. Their expression changes in the forebrain during postnatal development in rodents: GluN2B is first predominant and then declines two weeks after birth [4], [5]. GluN2A is weakly expressed at birth, rapidly increases at two weeks and then continues to rise progressively [5]–[7]. Since sensory deprivation retards this developmental shift, it was suggested that this shift is guided by experience [4], [5], [8], [9].

It is accepted that NMDARs become less plastic after development [10]. Nevertheless, electrophysiologically assessed changes in NMDAR after long term potentiation (LTP) induced by strong tetanic stimulation has been reported [11]. Later on, it was shown that the NMDAR subunits expression can be modified in adulthood by synaptic plasticity [10], [12]–[14], long term exposure to stressors [15] or corticosterone administration [16]–[18]. Grosshans *et al*. [19] reported an enhancement of GluN1 and GluN2A surface expression after LTP induction in mini-slices from adult rat hippocampus, with a concomitant decrease in intracellular levels. However, NMDAR expression after a behavioral experience is only scarcely documented [20], [21]. Steele *et al*. [22] reported that NMDA binding to chicken brain synaptosomal membranes increased after a passive avoidance task. Recently, Henderson *et al*. [20] reported that one and three weeks after training, there was an increase in cortical GluN1 and GluN2A NMDAR subunits.

Since the proposal of the NMDA-LTP hypothesis by Morris [23] and its involvement in memory processing, the relationship of NMDAR with synaptic plasticity (see [23]) and memory (see [21]) has been widely investigated. NMDAR membrane expression also changes immediately (seconds/minutes) after LTP induction by “tetanizing” high-frequency stimulation. Those changes were attributed to transport along the membrane from extrasynaptic sites and/or vesicular delivery from subsynaptic pools [10], [12], [14], [19], [24].

In this work our goal was to investigate if the expression of the major NMDAR subunits undergoes changes driven by behavioral experience and synaptic plasticity in adult rats. GluN1, GluN2A and GluN2B were analyzed by western blot or immunofluorescence in three different experimental models: 1) *in vivo* in adult rats that explored for 5 minutes an open field (OF), 2) *in vitro* in mature primary cultures of rat hippocampal neurons stimulated by KCl and 3) *in vitro* in fresh hippocampal slices from adult rats, where LTP was induced by theta burst stimulation (TBS). Similar modifications in NMDAR subunits were found in the three models. Then, we started to explore the putative mechanisms that would underlie those changes in hippocampal slices.

## Methods

### Behavioral Task

Male Wistar rats (180–250 g) were exposed to an OF (60.0 cm long × 40.0 cm wide × 50.0 cm high) for 5 minutes or just for 1 minute (as control for exposure to a new environment). The number of crossings from one quadrant (15.0 cm × 13.3 cm) to another, designed in the floor and the number of rearings *per* minute were recorded and compared to evaluate habituation to the environment. After the OF session, rats were sacrificed at different time points (0, 30 or 70 minutes), hippocampi were dissected and homogeneized for NMDAR subunits analysis by western blot.

Other rats were also left to explore the OF for either 1 or 5 minutes, and were exposed to the same arena either after 40 minutes or 24 hours (h) to assess short and long term memory (STM and LTM), respectively.

As controls of locomotion and exploratory behavior, NMDAR subunits were analyzed in the hippocampus of rats twice exposed for 5 minutes to the OF in two consecutive days and sacrificed 70 minutes after the second session.

All the procedures involving animals were carried out in accordance to the guidelines of the USA National Institutes of Health Guide for the Care and Use of Laboratory Animals and were approved by the Animal Care and Use Committees of the University of Buenos Aires.

### Primary Neuron Cultures, Culture Stimulation and Immunofluorescence

Hippocampal neuron cultures were performed as described by Kaech & Banker [25] with some modifications. In brief, both hippocampi were dissected from embryonic Wistar rats (E18) and digested with trypsin (Sigma, Sigma-Aldrich Co, MO, USA). Cells were plated onto poly-L-lysine (Sigma) -coated glass coverslips (Waldemar Knittel Glasbearbeitungs - GmbH, Germany) and incubated with D-MEM (Sigma), supplemented with 10% fetal bovine serum (FBS) (Natocor, Cordoba, Argentina). After 6 h, media was changed for Neurobasal (NB; Invitrogen, Life Technologies Corporation, CA, USA) supplemented with B27 (Invitrogen) and glutamine (Invitrogen) (complete NB). Cultures were maintained in complete NB at 37°C and 5% CO_2_.

Stimulation with 55 mM KCl was carried out as described by Wu *et al*. [26]. Cultures were fixed in paraformaldehyde 4%-sucrose 4% (Sigma) in phosphate buffer saline (PBS), immediately, 30 or 70 minutes after stimulation.

For immunofluorescence assays, coverslips were permeabilized in 0.1% Triton X-100 in PBS and blocked with 5% normal sheep serum-0.05% Tween-20 in PBS. Then, coverslips were incubated with anti-GluN1 (rabbit polyclonal, 1∶100, Sigma), anti-GluN2A (rabbit polyclonal, 1∶100, Chemicon, Millipore, MA, USA) or anti-GluN2B (rabbit polyclonal, 1∶100, Chemicon) antibody. Coverslips were then incubated with Cy2-conjugated anti-rabbit secondary antibody (1∶300, Jackson ImmunoResearch Laboratories, Inc., PA, USA).

### Image Analysis

Images from immunofluorescence assays were obtained under an Olympus-IX81 microscope and a FV300 Olympus Confocal microscope (Olympus CO, Tokyo, Japan). Images were analyzed by ImageJ software (ImageJ, NIH, Bethesda, MA, USA, http://imagej.nih.gov/ij/). Total immunofluorescence was quantified in 400X field images; immunofluorescence of each neuronal body was measured by ImageJ and plotted individually. To evaluate changes in NMDAR subunits at dendrites, 1000X images were used. Puncta were counted in isolated dendrites and were normalized to 10 µm. Each experiment was performed by triplicate.

### Electrophysiology

Hippocampal slices were prepared from Wistar rats (P42–60) using standard techniques [27]. Briefly, transverse slices from hippocampus (400 µm thick) were obtained with a vibratome (Electron Microscopy Sciences, EMS 5000) using chilled modified artificial cerebrospinal fluid (mACSF: 248 mM sucrose, 26 mM NaHCO_3_, 1.25 mM NaH_2_PO_4_, 5 mM KCl, 3 mM MgSO_4_, 1 mM CaCl_2_ and 10 mM D-glucose at pH 7.4), that was continuously bubbled with 5% CO_2_, 95% O_2_. Slices were then maintained at room temperature in an incubation chamber with regular artificial cerebrospinal fluid (ACSF: 125 mM NaCl, 26 mM NaHCO_3_, 1.25 mM NaH_2_PO_4_, 2.5 mM KCl, 1.3 mM MgSO_4_, 2.5 mM CaCl_2_ and 10 mM D-glucose at pH 7.4), continuously bubbled with 5% CO_2_, 95% O_2_. After at least 1 h incubation, slices were transferred to an immersion recording chamber perfused with ACSF at a 2 ml/min flow rate.

Both the stimulating tungsten bipolar electrode and the recording glass microelectrode filled with ACSF, were placed at CA1 *stratum radiatum* about 200 µm apart from each other. Field excitatory postsynaptic potentials (fEPSPs) were evoked by pulses at a 0.33 Hz stimulation frequency and with an intensity that was able to elicit a 50% the maximal fEPSP response. The fEPSP initial slope fEPSPs was analyzed. Baseline was determined after at least 30 minutes of stability. LTP was induced with a TBS protocol of three trains separated by 10 s. Each train consisted of 25 bursts of 4 pulses at 100 Hz, 200 ms apart (5 Hz). Drugs (40 µg/ml cycloheximide or 40 µM actinomycin D) were perfused from 3 minutes before TBS delivery until the end of the assay. At the end of the assay, each slice was homogeneized individually for western blot analysis.

### Western Blot (WB)

Each slice used in fEPSP recording assays, as well as both hippocampi from each animal exposed to the OF were separately homogenized in a Teflon glass potter (5×15′′) in 100 mM NaCl, 0.2% Triton X-100, 1 mM EGTA, antiproteases cocktail (Sigma), 20 mM HEPES (pH 7.4) buffer; and then incubated on ice 30 minutes to led to a complete lysis of the tissue. To avoid overloading the gel, protein concentration was preliminary estimated using the BCA kit (Sigma) in highly diluted (>100 folds) aliquots. In each experiment, to determine the actual amount of NMDAR subunits, the intensity of the NMDAR subunit band was relativized to the corresponding GAPDH band used as internal control. Samples were resuspended in Laemmli buffer and cracked at 100°C for 5 minutes. All samples were processed and analyzed individually. Protein samples were separated on a 10% SDS-PAGE gel and transferred to a polyvinylidenedifluoride membrane (Immobilon-P, Millipore). Blots were blocked with 3% non-fat milk-0.05% Tween-20 in Tris buffer saline (TTBS) and incubated with primary antibodies: anti-GluN1 (rabbit polyclonal 1∶1000, Sigma), anti-GluN2A (rabbit polyclonal, 1∶1000 Chemicon) or anti-GluN2B (rabbit polyclonal, 1∶1000 Chemicon); and anti-GAPDH (1∶5000, Sigma). After wash-out, blots were incubated with HRP-conjugated anti-rabbit secondary antibody (1∶2000; Amersham Biosciences, GE Healthcare, NJ, USA) or HRP-conjugated anti-mouse secondary antibody (1∶5000; Sigma), developed in SuperSignal West Pico Chemiluminescent Substrate solution (Thermo Scientific, MA, USA) and exposed to film (Agfa-Gevaert NV, Belgium).

### Statistical Analysis

Analysis was performed either by Student’s t test or by ONE WAY ANOVA, as indicated in the figures legends for each set of experiments. The ONE WAY ANOVA was followed by *post-hoc* analysis via Newman-Keuls or Dunnett tests when appropriate. All data are expressed as mean ± SEM. Behavioral data were analyzed using non parametric statistic (Kruskal Wallis Test or Mann Whitney) and expressed as medians with their interquartile ranges.

## Results

### 1.- Hippocampal NMDAR Subunit Levels after Exposure to a New Arena

**Figure 1:**
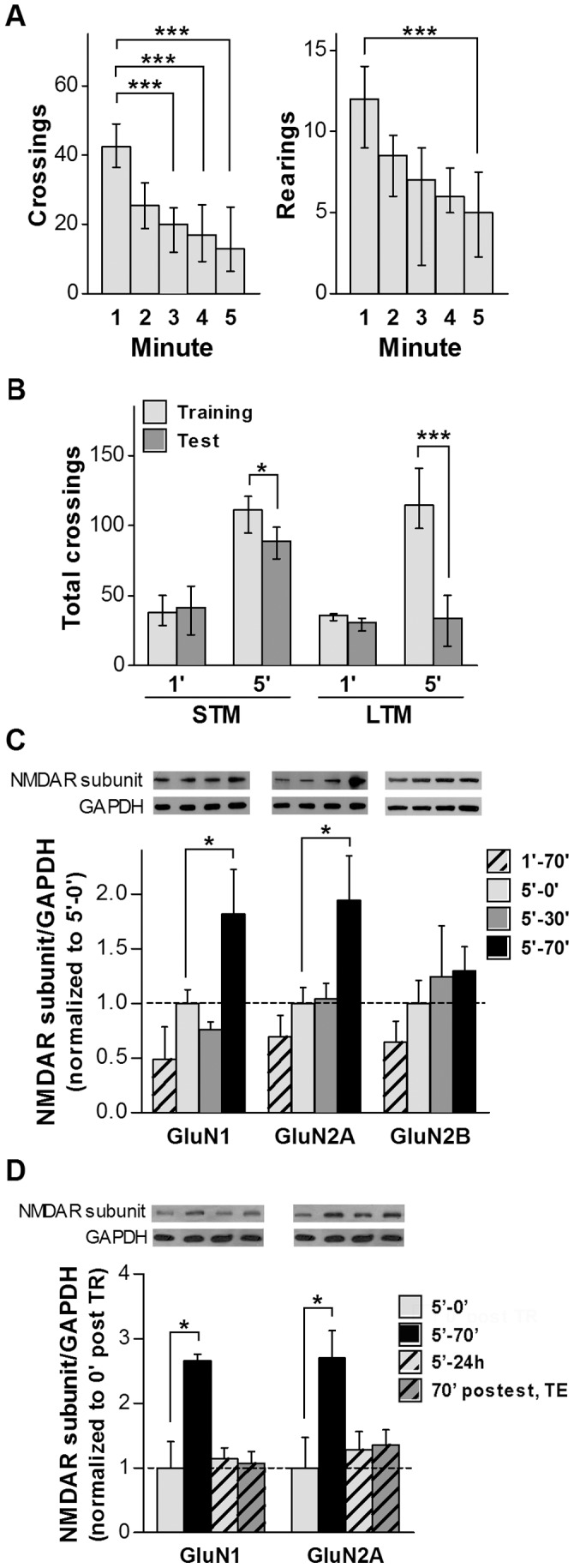
NMDAR subunits changes after OF habituation. *A.* Habituation to the OF of rats exposed to a 5 minutes OF session (n = 16). Graphs show number of crossings (left panel) and rearings (right panel) *per* minute (bars indicates median with interquartile ranges). Crossings decreased significantly after 3 minutes, while rearings were only significantly decreased in the fifth minute. *** p<0.0001, ** p<0.01 by Friedman test followed by Dunn’s Multiple Comparison Test. ***B.*** Total crossings from rats exposed to the OF for 1 or 5 minutes (Training) and tested for STM 40 minutes later (n = 12) or LTM 24 h later (n = 16). There were significant differences in total number of crossings in the second session compared to the first, only in rats which spent 5 minutes in the OF in the training session, for STM (* p<0.05) as well as for LTM (*** p<0.0001) (Mann Whitney test). ***C.*** NMDAR subunits in the hippocampus of rats after OF exposure. Four groups of rats were analyzed: rats as in ***A,*** which were sacrificed at 0, 30 and 70 minutes after the task (5′-0′, 5′–30′ and 5′–70′ groups); and rats exposed for 1 minute to the OF, sacrificed 70 minutes later (1′–70′ group). WB analysis showed about a one fold increase in GluN1 and GluN2A level for *5′–70′* group, in 3 independent experiments (* p<0.05, ONE WAY ANOVA, Newman-Keuls Multiple Comparison Post-Test). *Insert on top:* representative WB bands for GluN1, GluN2A and GluN2B NMDAR subunits and GAPDH (internal control). ***D***. NMDAR subunits analysis in the hippocampus of rats after two OF sessions. 4 groups of rats were analyzed: rats exposed to the OF 5 minutes and sacrificed immediately (5′-0′), 70 minutes (5′–70′), 24 h later (5′–24 h), or tested in the OF and sacrificed 70 minutes later (70′ postest-TE). * p<0.05 ONE WAY ANOVA, Dunnett’s Post-Test. *Insert on top:* representative WB bands for GluN1 and GluN2A NMDAR subunits and GAPDH (internal control).

It has been shown that 5 minutes but not 1 minute exploration of a new environment induces hippocampus dependent habituation, which persists as STM and LTM [28], [29]. In this work, rats were exposed to the OF for either 1 or 5 minutes. Habituation was assessed by counting and comparing number of crossings and rearings every minute. Rats which spent 5 minutes in the OF evidenced habituation since there was a significant decrease in the recorded exploratory parameters: the number of crossings were significantly lower in the third, fourth and fifth minute compared with the first minute in the OF, while the number of rearings decreased significantly in the fifth minute compared with the first minute (Figure 1A). Thus, it was corroborated that rats recognized the new environment showing habituation to it. Other rats were exposed to the OF for 1 or 5 minutes and were tested in a second OF session performed either 40 minutes or 24 h later, to evaluate STM or LTM respectively. The number of crossings were significantly lower in the second session only for those animals which were exposed for 5 minutes in the first session (* p<0.05, at 40 minutes; *** p<0.001 at 24 h; Mann-Whitney test) (Figure 1B). The total number of crossings for rats exposed for just 1 minute to the OF did not show any significant decrease when evaluated either 40 minutes or 24 h later (Figure 1B). These results showed that a 5 minutes OF session led to both STM and LTM formation and expression, while a 1 minute session did not.

Then, WB analysis of NMDAR subunits was carried out in hippocampal protein extracts of those rats exposed only once to the OF for 5 minutes (Figure 1C) and sacrificed at three different times: immediately after the OF session (5′–0′), 30 minutes (5′–30′) or 70 minutes (5′–70′) later (Figure 1C). WB analysis was also carried out in a fourth group corresponding to rats exposed once to the OF for 1 minute and sacrificed 70 minutes later (1′–70′).

Both GluN1 and GluN2A protein levels were significantly higher (about two fold) 70 minutes after 5 minutes in the OF (5′–70′), compared to 5′-0′, 5′–30′ and 1′–70′ groups, while GluN2B level did not show any significant modification (Figure 1C). There were not significant differences in NMDAR subunits between 5′-0′ and 1′–70′ groups. This result showing that there were no changes in the subunits of rats exposed only 1 minute to the OF (1′–70′), suggests that the rise in GluN1 and GluN2A subunits in the hippocampus of those rats which spent 5 minutes in the OF (5′–70′), would not be related to exposure to novelty.

To evaluate if habituation to a new environment, exploration or locomotion could be responsible for GluN1 and GluN2A changes, NMDAR subunits were analyzed in the hippocampus of rats twice exposed to 5 minutes OF sessions 24 h apart and sacrificed 70 minutes after the second session. These results were compared with those from rats exposed to a unique 5 minutes OF session and sacrificed either immediately, 70 minutes or 24 h later. As it is shown in Figure 1D, 70 minutes after the second 5 minutes session (Test), GluN1 and GluN2A levels were similar to those in 5′-0′ rats and in rats sacrificed 24 h after the OF, without a second OF session. Therefore, NMDAR subunits change observed 70 minutes after a single 5 minutes OF session was not observed in rats that explored twice the OF for 5 minutes each. These results confirmed that there were selective increases in hippocampal GluN1 and GluN2A subunits after a unique 5 minutes session in the OF and showed that these increases were transient since NMDAR subunits levels were similar to control rats in the following day (Figure 1D). Since rats exploring twice the same OF for 5 minutes have similar subunits levels than control animals, this strongly suggests that habituation, rather than just exploration or locomotion, would be related to the NMDAR subunits increase.

### 2.- NMDAR Subunits Change in Primary Cultured Neurons after KCl Stimulation

In order to find out whether similar changes like those observed after *in vivo* assays could take place in isolated neurons and to investigate where these changes would be localized, immunocytochemistry was carried out in mature hippocampal neuron cultures.

**Figure 2:**
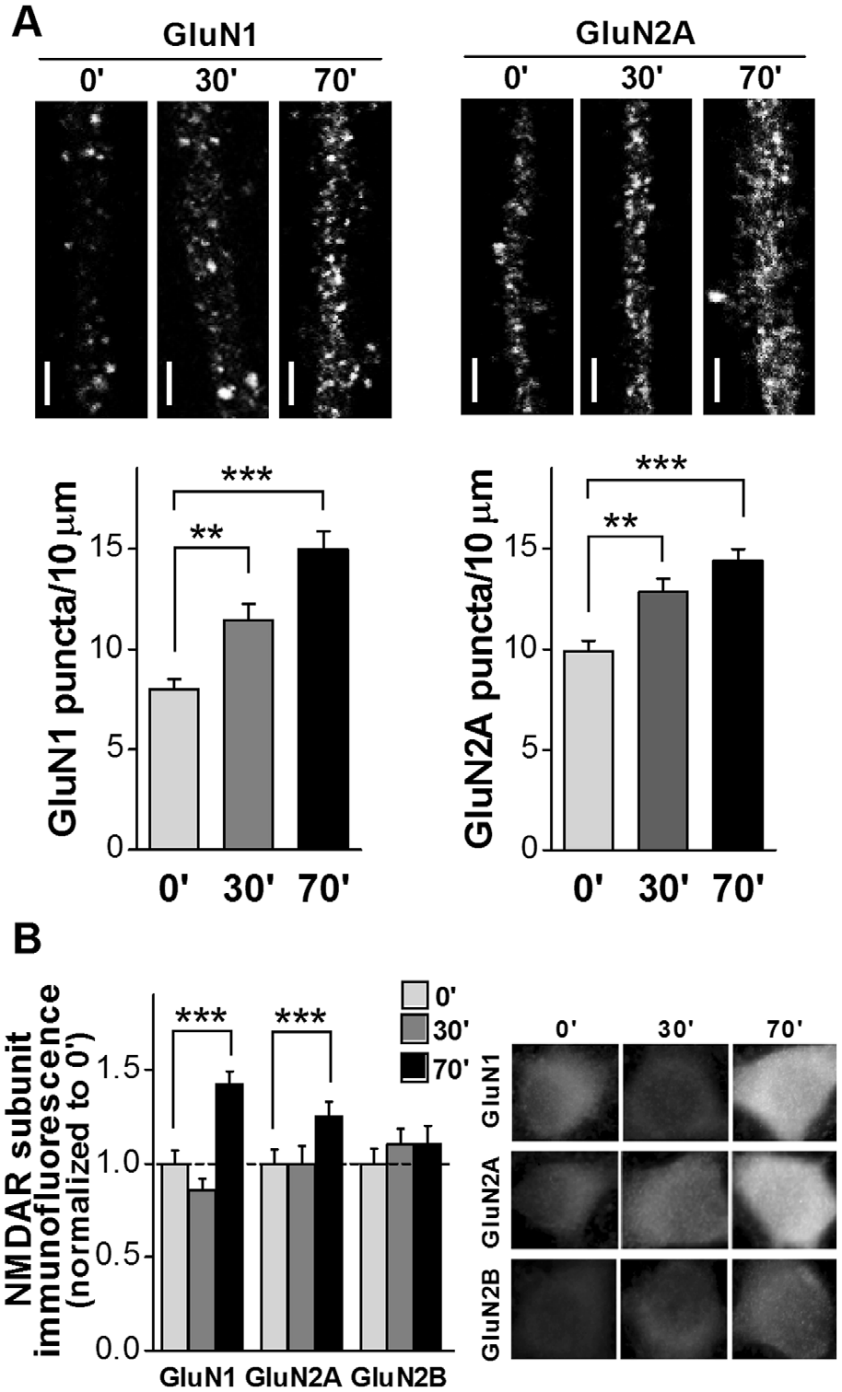
NMDAR subunits immunofluorescence in mature hipocampal neuron cultures stimulated by KCl. ***A.*** Quantification of NMDAR subunit puncta at dendrites (n = 100 neurites/culture). A significant increase in GluN1 and GluN2A puncta was observed at 30 and 70 minutes after KCl stimulation (** p<0.05, *** p<0.001, Kruskal-Wallis test followed by Dunn’s Multiple Comparison Post-Test). *Insert on the top of each bar*: representative dendrite for each condition (bar: 2 µm). ***B.*** Total fluorescence quantification 30 and 70 minutes after KCl stimulation. There were significant increases in GluN1 and GluN2A 70 minutes after stimulation. There were no significant changes in GluN2B total immunofluorescence (* p<0.05, *** p<0.001, ONE WAY ANOVA, Dunnet Post-Test). *Right:* representative neurons for each condition.

To induce “plastic-like” changes, repeated pulses of KCl were applied [26], [30]–[32]. First, it was verified that the already reported LTP-induced increase of NMDAR puncta in dendritic spines of hippocampal neurons [12], also took place in neurites in the KCl stimulated cultures. As it is shown in Figure 2A, GluN1 and GluN2A puncta increased significantly at neurites 30 and 70 minutes after KCl treatment, compared to controls fixed immediately after KCl treatment (Figure 2A, photos). There were about 1.5 and 2 fold increases of GluN1 puncta in neurites, 30 and 70 minutes after KCl pulses respectively (8±1 puncta/10 µm neurite in control cultures, 12±1 puncta/10 µm neurite in 30 minutes cultures and 15±2 puncta/10 µm neurite in 70 minutes cultures), indicating that a “plastic-like” change was already established in these neurons (Figure 2A).

GluN2 expression is required for GluN1 membrane expression [12]. Accordingly, after repeated depolarization by KCl there was also a significant increase of GluN2A puncta in neurites (13±1 and 14±1 puncta/10 µm neurite after 30 and 70 minutes, respectively, compared to 10±1 puncta/10 µm neurite immediately after depolarization [control]) (Figure 2A).

Then, total immunofluorescence was also assessed immediately (control), 30 and 70 minutes after KCl stimulation (Figure 2B). GluN1, GluN2A and GluN2B immunofluorescence in control cultures was not statistically different from cultures without stimulation (data not shown). Conversely, total immunofluorescence significantly increased for GluN1 (1.42±0.06 fold) and GluN2A (1.26±0.04 fold) 70 minutes after stimulation, compared to control cultures (Figure 2B). There was no significant difference in total immunofluorescence for any subunit 30 minutes after KCl stimulation. In addition, there were not significant changes in GluN2B total immunofluorescence at the times analyzed (Figure 2B).

These results indicate that changes in total immunofluorescence for each subunit in mature cultures are analogous to those observed after habituation to the OF. On the other hand, it must be noticed that the increase in puncta in neurites was already evident 30 minutes after stimulation, while there were not significant differences in total immunofluorescence.

### 3.- GluN1 and GluN2A but not GluN2B, Increase after LTP Induction and Expression

**Figure 3:**
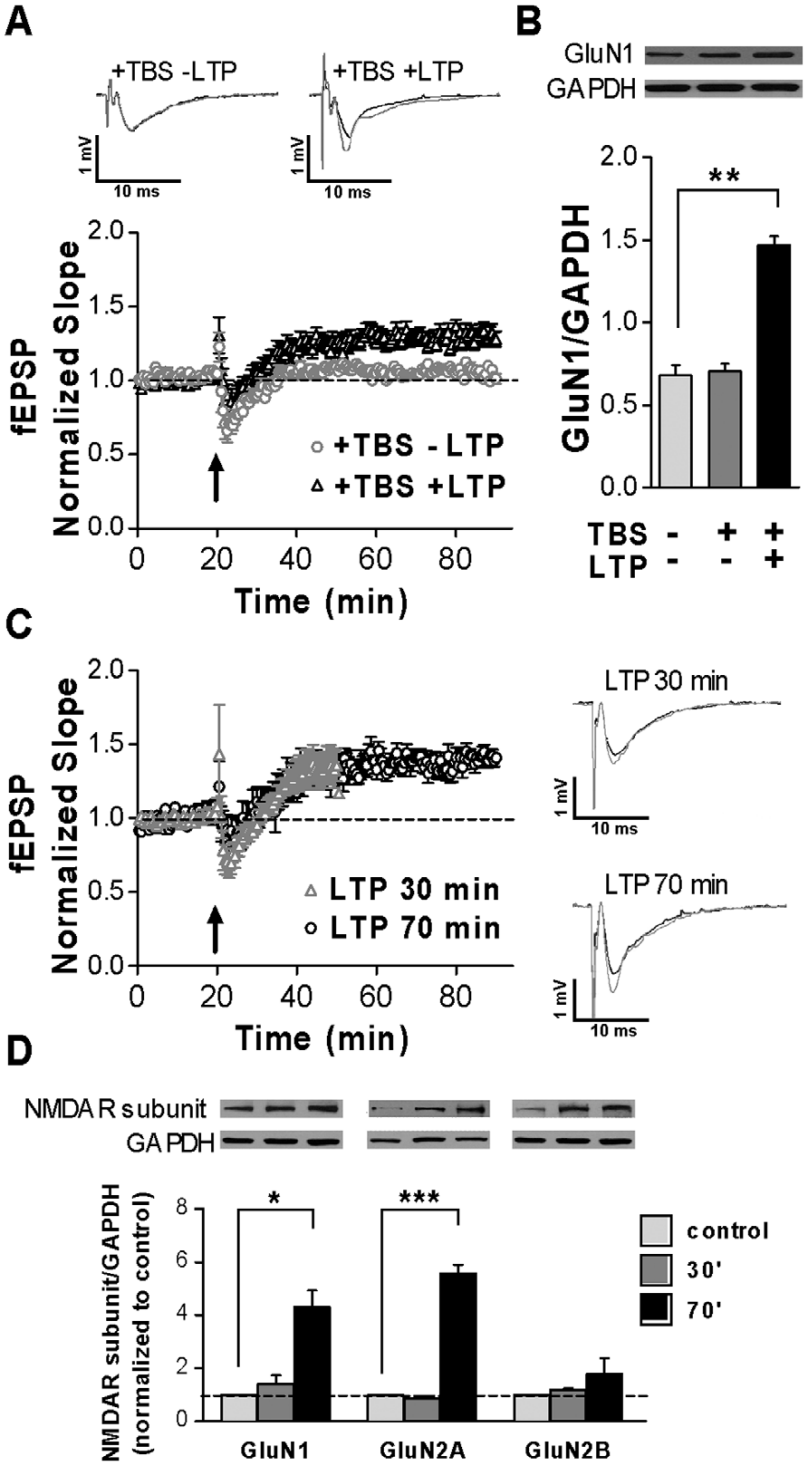
NMDAR subunits change after LTP induction and expression. ***A.*** Evoked fEPSPs normalized slopes from fresh hippocampal slices corresponding to the first pulse of paired stimulation, before and after TBS (arrow). Plots represent the average of three independent experiments over 90 minutes of recording (n = 6 for each group). *Insert on top:* average traces of 10 individual recordings from a +TBS+LTP and a +TBS-LTP slices (black: 5 minutes before TBS; grey: 5 last minutes of recording). ***B.*** WB band densities quantification of samples from same slices that in **A.** A significant increase was only observed for +TBS+LTP slices (** p<0.01; *** p<0.001 ONE WAY ANOVA, Dunnet Post-Test; n = 6 for each group). *Insert on top:* (from left to right): representative GluN1 and GAPDH WB bands from: a −TBS slice, a +TBS-LTP slice and a +TBS+LTP slice. ***C.*** Evoked fEPSPs slopes corresponding to the first pulse of the paired stimulation before and after TBS (arrow). Plots represent the average of fEPSPs slopes over 50 and 90 minutes of recording, respectively (n = 6 for each group). *Right*: average traces of 10 individual recordings from a LTP-slice after 30 and 70 minutes TBS (black: 5 minutes before TBS; grey: 5 last minutes of recording). ***D.*** NMDAR subunits quantification by WB. Samples analyzed: slices used in **C**. (processed 30 or 70 minutes after TBS) and in −TBS slices (Control). Analysis of WB bands showed a significant increase in GluN1 and GluN2A level for the 70 minutes group in three independent experiments (* p<0,05; *** p<0,001 ONE WAY ANOVA-Dunnet Test). *Insert on top:* Representative WB bands for GluN1, GluN2A and GluN2B NMDAR subunits and GAPDH (internal control).

NMDAR subunits were analyzed after LTP induction at CA1 synapses in fresh hippocampal slices from adult rats. WB analysis for GluN1 was performed in the following samples: 1) fresh slices only stimulated with 0.33 Hz pulses over 100 minutes to evoke fEPSPs (−TBS) (Figure 3B); 2) fresh slices receiving TBS, where LTP was effectively induced as corroborated by recordings performed over the following 70 minutes (+TBS+LTP) (Figure 3A and B); and 3) slices under similar conditions as those in (2), but where TBS failed to induce LTP (+TBS-LTP) (Figure 3A and B). A protein extract from slices without any treatment was also analyzed by WB.

As it is shown in Figure 3B, there was a significant increase of about 50% in GluN1 band density, assessed 70 minute after TBS delivery in +TBS+LTP slices compared to −TBS slices or +TBS-LTP slices. In addition, results from −TBS slices were not significantly different from +TBS-LTP slices (Figure 3B) or from slices without any treatment (data not shown).

In an independent set of experiments, GluN1 and both GluN2 subunits were quantified either at 30 or 70 minutes after LTP induction (Figure 3C and D). There was an increase in GluN2A (6.1±0.5 fold) and in GluN1 levels (4.7±2.2 fold), in +TBS+LTP slices compared to −TBS slices, assessed 70 minutes after TBS delivery (Figure 3D). However, there were not significant changes in these subunits 30 minutes after TBS (Figure 3D). In the same slices, GluN2B bands were not significantly different compared to −TBS slices (Figure 3D).

In accordance with *in vivo* and *in vitro* results reported above (Results sections 1 and 2), there were significant increases of both GluN1 and GluN2A subunits but not in GluN2B at 70 minutes, while there were no significant changes in total levels of NMDAR subunits 30 minutes after induction of plasticity.

### 4.- What are Changes in GluN1 and GluN2A Levels in Hippocampal Slices Depending on?

To start to investigate if transcription and/or translation could be involved in the NMDAR subunit changes observed after LTP induction, fresh hippocampal slices were treated either with the translation inhibitor cycloheximide (CHX) or with the transcription inhibitor actinomycin D (ActD). Electrophysiological assays in slices perfused either with ActD or CHX and WB analysis (see Results section 3) were carried out.

**Figure 4:**
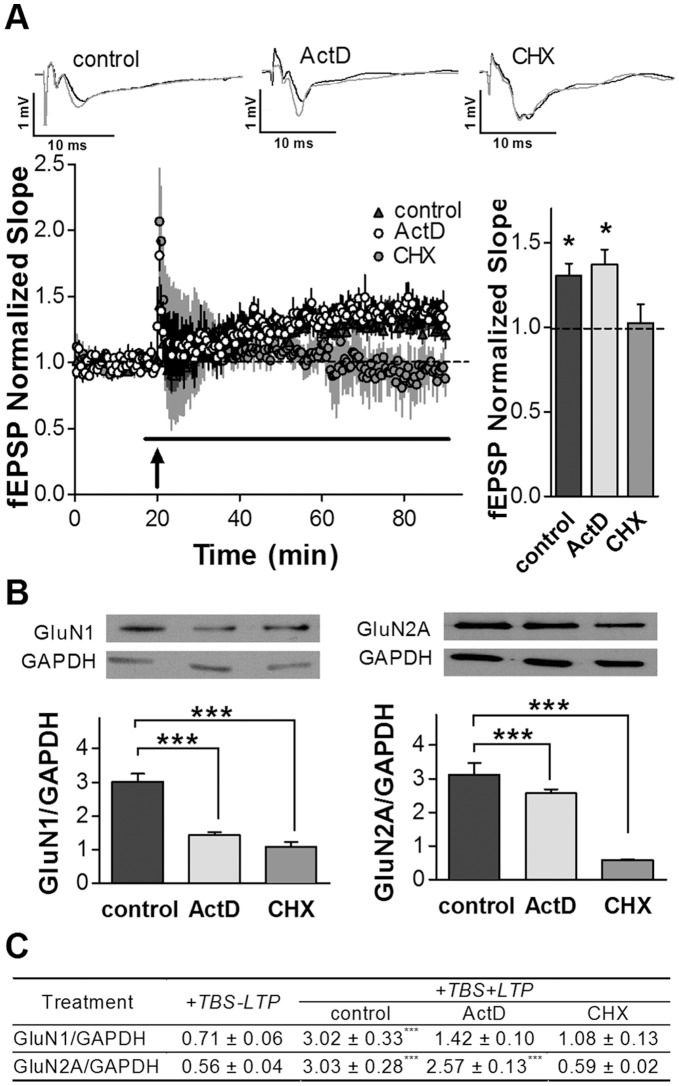
NMDAR subunits modification in hippocampal slices after transcription or translation inhibition during LTP induction. *A.* * Left:* Normalized slopes of evoked fEPSPs recorded as those in Figure 3, corresponding to the first pulse of the paired stimulation applied before and after TBS (arrow). Plots represent the average of five independent experiments over 90 minutes of recording (n = 5 for each group). *Black line*: drug perfusion. *Insert on top*: average traces of 10 individual recordings from a control slice and slices treated with ActD or CHX (black: 5 minutes before TBS; grey: 5 last minutes of recording). *Right:* Bars represent averages of normalized first pulse slopes of the 5 last minutes of recording for each group. LTP induction was blocked by 40 µg/ml CHX treatment (* p<0.05, one sample t test) compared to basal transmission (line referred to 1 in the graph). ***B.*** NMDAR subunits were evaluated by WB in same slices that in **A.** CHX treatment blocked GluN1 and GluN2A increase, while 50 µg/ml ActD only blocked GluN1 increase (* p<0,05 ONE WAY ANOVA, Dunnet Post-Test; n = 5 for each group). *Insert on top:* (from left to right): Representative GluN1 and GluN2A WB bands of +TBS+LTP slices (control), CHX and ActD +TBS+LTP slices treated slices. ***C.*** Table indicates mean ± SEM for GluN1/GAPDH (first row) or GluN2A/GAPDH (second row) in +TBS-LTP slices (n = 6) and +TBS+LTP slices without any drug treatment (Control in **B,** n = 9), or treated with ActD (n = 5) or CHX (n = 5) (*** p<0.0001; ONE WAY ANOVA - Newman Keuls Test).

Slices perfused with 40 µM ActD developed LTP after TBS induction (Figure 4A). In contrast, LTP was not effectively induced by TBS in slices perfused with CHX (Figure 4A). This result is in agreement with previous reports showing that LTP is a translation-dependent process [14], [33]–[36]. In CHX perfused slices, there was neither a significant increase in GluN1 nor in GluN2A after TBS (Figure 4B). These results indicate that in the hippocampal slices, the observed changes in both subunits depend on translation mechanisms. In addition, since the subunits appeared to remain unchanged in those slices that received TBS but did not develop LTP (+TBS-LTP slices; Figure 4C), our results suggest that the modifications would be related to LTP induction and expression.

In slices treated with 40 µM ActD, in spite of an effective LTP induction and expression for at least 70 minutes (in agreement with previous reports [14], [37]), GluN1 level did not increase after TBS, being not significantly different from that in +TBS-LTP slices (Figure 4C); whereas the GluN2A band density was as high as in +TBS+LTP slices (without any drug treatment) (Figure 4B and C). Hence, at least with the concentration of ActD and the conditions used here, GluN1 increase was blocked while GluN2A increase was not affected. Therefore, as it is shown in Figure 4C, GluN1/GAPDH ratio did not significantly increase in +TBS+LTP slices treated with either ActD or CHX.

Altogether, these results suggest that both, transcription and translation appear to be involved in GluN1 rise after TBS. On the other hand, GluN2A/GAPDH ratios in +TBS+LTP slices without any drug or treated with ActD were significantly higher than those in CHX treated slices, strongly suggesting that translation would be responsible for TBS-LTP-GluN2A induced increase (Figure 4C).

## Discussion

### 1.- Hippocampal GluN1and GluN2A Proteins Increase in vivo after 5 Minutes in the OF

NMDARs antagonists administered after training caused retrograde amnesia for habituation as well as for other spatial and aversive memories [21], [23], [38]. Packard and Teather [39] had reported that NMDARs inhibition up to 2 h after training, though no later, impaired consolidation of spatial memory.

There is only scarce information concerning NMDAR subunits differential participation in learning and memory. Overexpression of GluN2B subunits led to facilitation of LTP induction and to an improvement in spatial memory [40]. However, after selective blockade of GluN2B containing receptors, Guscott *et al*., [41] did not observe any effect on learning and memory; whereas Ge *et al*. [42] have shown that the GluN2B blockade inhibited long term depression (LTD) and impaired spatial memory in freely moving rats, suggesting a crucial role for these receptors in memory consolidation. These results also confirmed those obtained with mutants mice with GluN2B deletion in CA1 hippocampal and cortical pyramidal neurons [43]; there was a loss of LTD, a partial deficiency of LTP (a threshold increase) and profound cognitive deficits on hippocampal dependent tasks. GluN2B deletion in CA1 cells and dentate gyrus granule cells impaired some (spatial reversal and working memory) but not all (spatial reference) hippocampus dependent learning [44]. On the other hand, GluN2A deletion impaired performance in some hippocampal dependent tasks [45]. Thus, it was proposed that the combined loss of GluN2B and GluN2A (or GluN1 *per se*) in CA1 is necessary to produce memory deficits in some spatial learning tasks [43].

As it was previously reported [28], [29], rats exposed during 5 minutes to an OF showed spatial habituation, which persisted as STM as well as LTM. Here we showed that after a unique 5 minutes session in the OF –though not after a 1 minute session-, there was an increase in GluN1 and GluN2A subunits. These results indicate that just the exposure to novelty –1 minute session- was not able to elicit those changes. Habituation were assessed and corroborated minute after minute in the first 5 minute session, and in a second session performed 40 minutes or 24 h later to evaluate STM and LTM, respectively. Since rats exposed twice to the OF in two consecutive days did not show statistically significant modifications in the NDMAR subunits analyzed, this strongly suggests that habituation, rather than just exploration or locomotion, was related to those changes and shows that they were transient. Further investigation is necessary to find out the possible relevance of subunits increase for learning and memory.

### 2.- GluN1 and GluN2A Increase in Cultured Neurons after KCl Stimulation

LTP induces NMDAR mobility from cell body and dendrites to the synapses, as revealed by an increase in NMDAR puncta in dendritic spines [12], [46]. Here we showed that after repeated depolarization of hippocampal neuron cultures, GluN1 and GluN2A puncta significantly increased in neurites when assessed 30 and 70 minutes after stimulation. Total immunofluorescence for both subunits increased at 70, but not at 30 minutes. Therefore, differential transport from the cell body and the subsynaptic pool [10], [12], [14] would account for the 30 minutes increase in puncta in neurites, since each subunit total amount appeared to remain unchanged at this time. On the other hand, the increase in total immunofluorescence at 70 minutes would require protein synthesis. The increase in the corresponding puncta strongly suggests that new NMDARs containing GluN2A subunits have been assembled and expressed in the surface both at 30 and at 70 minutes. Accordingly, after 30 minutes of LTP induction, Grosshans *et al*. [19] reported an intracellular decrease and a surface increase of both, GluN1 and GluN2A subunits.

In mature neuron cultures (22–30 days *in vitro*), NMDAR activity tonically suppressed GluN2B translation, while long-lasting pharmacological blockade of NMDAR released that suppression [47]. Hence, our results are in agreement with the fact that neuronal activity facilitates GluN2A expression and suppresses GluN2B translation in mature neurons, both leading to an increase in GluN2A/GluN2B ratio in response to neural activity.

### 3.- GluN1 and GluN2A Proteins Increase after LTP Induction in Hippocampal Slices

GluN1 and GluN2A but not GluN2B, significantly increased after LTP effective induction in adult rat hippocampal slices. Since the amount of NMDAR subunits in slices without TBS was not different from that in slices that did not express LTP after TBS (+TBS-LTP), it can be concluded that LTP effective induction is required for the subunits increase. The results obtained in hippocampal slices are coherent with the increases observed in NMDAR subunits both *in vivo* in rats and *in vitro* in neuron cultures. Grosshans *et al*. [19] reported an enhanced GluN1 and GluN2A surface expression 15 and 30 minutes after LTP induction in CA1 mini-slices from adult rat; since the intracellular subunits levels concomitantly decreased, they proposed that GluN1 and GluN2A were recruited from available pools and suggested that this could represent a persistent postsynaptic modification since the change was present after 180 minutes. Accordingly, in hippocampal slices we did not find any significant change in subunits level at 30 minutes, though in the neurons culture there was an increase in puncta at neurites. In addition, here we reported a significant increase of both subunits at 70 minutes that could account for a long term modification.

NMDAR activation mediates α-amino-3-hydroxy-5-methyl-4-isoxazolepropionic acid receptor (AMPAR) membrane insertion and this was proposed as a main mechanism for hippocampal NMDAR-dependent LTP. Interestingly, NMDAR activation has differential effects on AMPAR trafficking depending on its subunit composition: in cultured neurons, GluN2A promoted whereas GluN2B inhibited surface expression of AMPARs [48].

### 4.- GluN1 and GluN2A Increases in Hippocampal Slices Depend on Different Mechanisms

Transcriptional and translational regulation of NMDAR subunits has been mostly investigated during early postnatal development in rodents. In early postnatal stages, brain stem, hippocampus and neocortex showed enhanced *glun2a* transcription, which was proposed to be driven by activity-dependent activation of GluN2B-containing NMDARs; this enhanced expression increases the GluN2A/GluN2B ratio [46], [49].

Translation and transcription can be separated mechanisms in neurons. Some mRNAs can be stored in the cytoplasm as ribonucleoparticles (RNPs). Some of these RNPs are stored and are translated only when an appropriate stimulus arrives [1], [21], [50].

We have shown that after perfusion of hippocampal slices with CHX there was neither increase in GluN1 nor in GluN2A, and there was not LTP expression following TBS delivery. The latest was expectable as it was already shown that both memory acquisition and LTP induction are translation dependent processes [33]–[36], [51]–[53]. Hence, our results corroborated that LTP induction requires protein synthesis and indicated that translation and LTP effective induction are required for the increase in NMDAR subunits.

Yin *et al*. [14] reported that late LTP (L-LTP) in slices from mice was inhibited, though with distinct kinetic profiles, by both anisomycin and ActD. They showed that perfusion of 40 µM ActD 30 minutes before high frequency stimulation (HFS), did not seem to produce modifications in potentiation until about 75 minutes after HFS; however, L-LTP started to decrease later on; this inhibition became statistically significant at about 210 minutes after induction [14]. Hence, it was proposed that this early LTP (E-LTP) or even the “early steps of L-LTP” were independent on transcription [14], [37]. Accordingly, in our experiments with the same ActD concentration, LTP was effectively induced and its expression persisted for at least 70 minutes after TBS. It was shown that ActD rapidly inhibited the induction of transcription (i.e. suppressing BDNF-induced upregulation of Arc [54]). Although we cannot fully discard some remaining transcriptional activity during ActD perfusion, GluN1 increase was blocked while GluN2A increase was not affected with the concentration of ActD used in this work. Hence, we have shown that the rise in GluN2A in the slices seems to mainly depend on translation, while at present we cannot discard some transcriptional contribution. On the other hand, GluN1 increase would depend on both transcription and translation mechanisms.

Therefore, NMDAR subunits increase after LTP induction in hippocampal slices requires protein synthesis. Although this increase in translation -and may be in transcription- might be interpreted as a consequence –instead of a cause- of the subunits synaptic recruitment, the levels remained significantly higher than controls, suggesting that, at least for a while, a new steady state could have been reached.

Our results indicate that translation of already transcribed mRNAs was necessary. Although gene expression appeared not to be required for LTP induction and expression over at least 70 minutes (E-LTP?) [14], [54], with the actual data we could not discard the contribution of some remaining transcription during ActD perfusion. Since a GluN1 pool is retained in the endoplasmic reticulum (ER) [55], [56], NMDAR could still increase at the surface without *de novo* expression of GluN1, whenever GluN2 subunits are available [12]. Changes in NMDAR would be –at least partially- supported by GluN1 present in ER and translation of GluN2A from already transcribed mRNAs [21], [55]. *In silico* analysis of GluN2A mRNA revealed that there are 6 upstream open reading frames (uORFs) present in *glun2A* 5′UTR [57]. This is a known regulatory mechanism of translation which might be responsible for GluN2A mRNA ordinary translation at a slow rate in non-active neurons and of its translation enhancement after certain synaptic stimulus (when only specific plasticity-related mRNAs would be translated).

### 5. NMDAR Subunits Undergo Similar Changes in the Experimental Models Analyzed

In the three models used, GluN1 and GluN2A increased after 30 and before 70 minutes following induction of plasticity or exposure to the OF, whereas no changes were observed in GluN2B. This happened *in vivo*, in adult rats, and *in vitro* both in hippocampal slices of adults and in mature cultures of hippocampal neurons. Although these results are similar, further investigation is required to reveal if the mechanisms involved in each case reflect related processes and to find out whether they are causally related with synaptic plasticity, learning and memory.

Changes in NMDAR subunits were first reported during early post-natal development in mammals [4], [51], [58], when NMDAR subunit expression switches from GluN2B-containing receptors to GluN2A-receptors predominance [21]. These changes are slowly developed over the course of days and are dependent on transcription, translation and activity [12]. In rat hippocampal slices different activity-dependent mechanisms regulate synaptic delivery of each NMDAR subunit. In general, activity would lead to an increase in GluN2A and a decrease in GluN2B synaptic membrane expression, as was assessed by changes of currents kinetic in voltage-clamp recordings in organotypic cultures or in fresh slices [10], [12], [14], [24], [59]. These increases in GluN2A/GluN2B ratio occured very quickly and seemed to be independent of either protein synthesis or gene expression [10], [24].

GluN2B-NMDARs appear to be necessary for LTP induction, with higher affinity for Calcium/Calmodulin-Dependent Protein Kinase II (CaMKII) than GluN2A [60] and higher capacity to carry two fold the charge for a single synaptic event than GluN2A-containing receptors [61],[62]. However, it was reported that the selective blockade of GluN2B-NMDARs abolished the induction of LTD but not of LTP [42], [43]. In contrast, preferential inhibition of GluN2A-NMDARs prevented LTP induction [63]. But Foster *et al*. [59] suggested that an excess of GluN2A could inhibit LTP induction in cultured slices. Furthermore, it was reported that activation of GluN2B-NDMARs (but not of GluN2A) leads to excitotoxicity and cell death [64]. Thus, an increase in GluN2A/GluN2B ratio could likely be neuroprotective.

### Concluding Remarks and Perspectives

Here we showed that after a novel experience leading to habituation (*in vivo*), as well as after TBS-LTP induction in hippocampal slices and following KCl stimulation in neurons culture (*in vitro*), there was a significant increase in both GluN1 and GluN2A levels. The rise in GluN1 following LTP induction in slices depends on transcription and translation, whereas the concomitant rise in GluN2A seems to mainly depend on translation, though the contribution of putatively remaining transcription during ActD perfusion could not be ruled out. In the presence of 40 µM ActD, LTP was established for at least the first 70 minutes.

LTP effective induction is required for the increase in NMDAR subunits. Translation from pre-existing mRNAs is required for LTP induction and expression.

Our results open several questions about the functional significance of such NMDAR subunits changes: Are rises in both GluN1 and GluN2A causally related to synaptic plasticity establishment/persistence, i.e., contributing to the transition from E-LTP to L-LTP in a time well after the induction? Are these changes related to later phases of memory storage/consolidation, leading to LTM or to later plasticity involved in memory persistence? Could an increase in GluN2A/GluN2B ratio be a compensatory/homeostatic consequent adaptation once a synapse undergoes a plastic change like potentiation, i.e., by decreasing the probability of further synaptic plasticity or even preventing excitotoxicity?
